# Synthesis of Ge_1−x_Sn_x_ Alloy Thin Films by Rapid Thermal Annealing of Sputtered Ge/Sn/Ge Layers on Si Substrates

**DOI:** 10.3390/ma11112248

**Published:** 2018-11-12

**Authors:** Hadi Mahmodi, Md Roslan Hashim, Tetsuo Soga, Salman Alrokayan, Haseeb A. Khan, Mohamad Rusop

**Affiliations:** 1 Nano-Optoelectronics Research Laboratory, School of Physics, Universiti Sains Malaysia, 11800 USM Pulau Penang, Malaysia; 2 Institute of Nano-Optoelectronics Research and Technology Laboratory, Universiti Sains Malaysia, Penang 11900, Malaysia; roslan@usm.my; 3 Department of Electrical and Mechanical Engineering, Nagoya Institute of Technology, Nagoya 466-8555, Japan; soga.tetsuo@nitech.ac.jp; 4 Research Chair for Biomedical Applications of Nanomaterials, Biochemistry Department, College of Science, King Saud University, Riyadh 11451, Saudi Arabia; salrokayan@ksu.edu.sa (S.A.); haseeb@ksu.edu.sa (H.A.K.); 5 NANO-SciTech Centre, Institute of Science, Universiti Teknologi MARA, Shah Alam, Selangor 40450, Malaysia; nanouitm@gmail.com

**Keywords:** semiconductors, thin films, Ge-Sn, nanocrystalline, sputtering, Raman spectroscopy, scanning electron microscopy, X-ray diffraction

## Abstract

In this work, nanocrystalline Ge_1−x_Sn_x_ alloy formation from a rapid thermal annealed Ge/Sn/Ge multilayer has been presented. The multilayer was magnetron sputtered onto the Silicon substrate. This was followed by annealing the layers by rapid thermal annealing, at temperatures of 300 °C, 350 °C, 400 °C, and 450 °C, for 10 s. Then, the effect of thermal annealing on the morphological, structural, and optical characteristics of the synthesized Ge_1−x_Sn_x_ alloys were investigated. The nanocrystalline Ge_1−x_Sn_x_ formation was revealed by high-resolution X-ray diffraction (HR-XRD) measurements, which showed the orientation of (111). Raman results showed that phonon intensities of the Ge-Ge vibrations were improved with an increase in the annealing temperature. The results evidently showed that raising the annealing temperature led to improvements in the crystalline quality of the layers. It was demonstrated that Ge-Sn solid-phase mixing had occurred at a low temperature of 400 °C, which led to the creation of a Ge_1−x_Sn_x_ alloy. In addition, spectral photo-responsivity of a fabricated Ge_1−x_Sn_x_ metal-semiconductor-metal (MSM) photodetector exhibited its extending wavelength into the near-infrared region (820 nm).

## 1. Introduction

Silicon (Si) has been the dominant semiconductor material for about a few decades. The introduction of Ge_1−x_Sn_x_ alloys [1] has extended the dominance of Si technology into areas previously dominated by III-V materials [2]. However, Si, Ge, and Ge_1−x_Si_x_ are indirect band-gap semiconductors and, therefore, cannot be used to fabricate laser, since it requires a direct band-gap material. Recently, Ge_1−x_Sn_x_ alloy, as a group-IV alloy semiconductor, has received significant attention due to the potential possibilities for advanced optoelectronic devices, in future [3,4]. GeSn offers tunable direct band-gap when the Sn content is larger than about 6.5%–11% [5,6,7]. In addition, this alloy band gap can be tuned in the infrared range. In recent years, several types of optoelectronics and electronics devices such as laser [8], light emitting diodes (LEDs) [9], diodes [10], photoconductors [11], and p-i-n photodetectors [12] have been fabricated from GeSn film.

The low solubility of Sn and Ge (>1%) [13], Sn surface segregation [14] and huge lattice mismatch between α-Sn and Germanium [15] make the growing of epitaxial Ge_1−x_Sn_x_ films difficult and introduce more challenges. However, non-equilibrium growth approaches have been developed for growing GeSn alloys; including molecular beam epitaxy (MBE) [16,17], chemical vapor deposition (CVD) [18,19,20], solid phase epitaxy (SPE) [21], pulsed laser deposition (PLD) [22], and magnetron sputtering [12,23,24]. Consequently, high-quality epitaxial Ge_1−x_Sn_x_ alloy film have been grown by these techniques. Compared to the above-mentioned growth methods, magnetron sputtering provides some advantages, such as a low-cost technique, an independent and easily controlled growth rate and growth temperature, a simple control over the alloy composition, and most significantly, the Ge and Sn targets are much safer than the currently used precursor gas, in an MBE or CVD. In addition, these techniques require a precise substrate temperature control and very clean Si surface. Up till now, limited research has been carried out on the use of sputtering, to grow a GeSn alloy. Growing crystalline GeSn layers, by post-deposition annealing, has also not been well-studied. Through this technique, epitaxial GeSn films are formed by depositing an amorphous GeSn film on the Si or Ge substrate, followed by an annealing process. The driving force for the GeSn formation is the incorporation of the Sn into the Ge, through a solid-phase mixing. The post-deposition methods that have been used to crystalize GeSn alloy consist of a solid-phase epitaxy, thermal, and laser annealing. The solid phase crystallization temperature of GeSn is lower than those of the Ge and Si_1−x_Ge_x_. The annealing temperature for SiGe annealing, using this technique, requires over 1000 °C to mix the germanium and the silicon [25,26,27]. However, the employment of this technique for GeSn formation is desirable, due to its simple process and low cost.

In this study, the multilayers of Ge/Sn/Ge were sputtered onto Si substrates, from high purity targets. Then, the layers of the multilayer structure were rapid thermal annealed (RTA), at various temperatures, over a short duration. The findings of this research present the effect of thermal annealing on the morphological, structural, and optical characteristics of the synthesized GeSn alloy layers, on Si. It was observed that utilizing the higher annealing temperatures resulted in an improvement of the crystallinity and optical characteristics of the nanocrystalline layers. In this experiment, the Ge-Sn solid-phase mixing (inter-diffusion) was a main driving force for the GeSn alloy formation, through rapid thermal annealing.

## 2. Materials and Methods

Prior to the deposition process, the RCA (Radio Corporation of America) cleaning technique was used to clean the n-type Si (100) wafers. The multilayer structure was obtained by subsequent deposition of Ge and Sn layers, onto the Si substrate. The amorphous multilayer, Ge/Sn/Ge, were sputtered in a radio frequency (RF) magnetron sputtering system (Edwards A500). The background pressure of the chamber was 1.20 × 10^−5^ mbar and high purity Ar (99.999%) was used as a sputtering gas. The diameter of Germanium (99.999%) and the Tin (99.999%) targets were 10 cm and were placed 10 cm beneath the sample holder. The magnetrons were placed in a planar configuration. The multilayer was sputtered at a Ge RF power of 100 W and Sn RF power of 15 W, at room temperature (RT). After deposition, multilayers were rapid thermal annealed at 300 °C, 350 °C, 400 °C, and 450 °C, for 10 s, in the nitrogen ambient.

**Figure 1:**
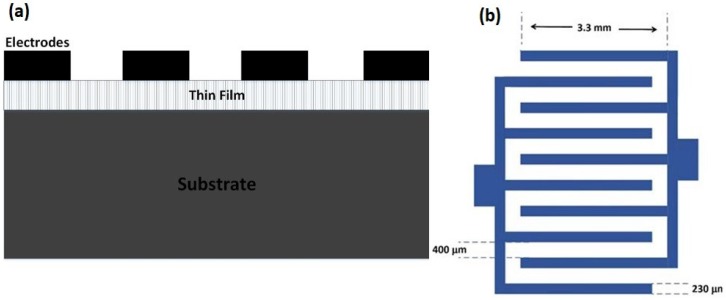
(**a**) Schematic of the metal-semiconductor-meal photodetector (MSM PD); and (**b**) schematic of the metal contact for the MSM PD fabrication.

To fabricate a metal-semiconductor-meal photodetector (MSM PD), Nickel was deposited onto the GeSn thin films, via vacuum thermal evaporation, to make two interdigitated Schottky contacts, through a metal mask (fingers pattern). Schematic of the MSM photodetector is shown in Figure 1a. In addition, as shown in Figure 1b, each electrode had five fingers. The width and length of each finger were 230 μm and 3.3 mm, respectively. The spacing between each finger was 400 μm. The fabricated devices were annealed at 400 °C, in the tube furnace, with nitrogen flowing for five minutes. The thickness of the metal contacts was about 200 nm.

The surface morphology of the samples was characterized via Field emission scanning electron microscopy (FESEM) (Nova NanoSEM 450, FEI, Netherlands). Energy-dispersive X-ray spectroscopy (EDX) was employed to recognize the elements existing in the films at 10 KV acceleration voltage. Atomic force microscopy (AFM) (Dimension edge, Bruker, Billerica, MA, USA) was used to acquire AFM images, with a non-contact operation mode and a Nano Drive dimension-edge-tapping image-processing software (Version 6.13). Raman measurement was carried out with a Jobin-Yvon (HR800) spectrometer (Horiba, Longjumeau, France), where the thin films were excited at room temperature (RT) with an argon ion laser (514.5 nm, 20 mW). The crystallographic characterization of the layers was analyzed using a high-resolution X-ray diffractometer (HR-XRD) system (X’Pert3040) (Panalytical, Malvern, UK). The electrical measurements of the MSM photodetector were performed at RT, with a computer-controlled integrated SourceMeter Instrument (Model Keithley 2400, USA).

For the spectral responsivity measurement of the Ge_1−x_Sn_x_ MSM PD, the combination of a Xenon lamp and monochromator were used to generate the spectrum of light, with different wavelengths. The spectral responsivity of an optical detector is a measure of its electrical response to optical radiation, at a specified wavelength, which can be given as:

$$R=\frac{I_{\mathrm{photo}}-I_{\mathrm{dark}}}{E}$$

where I_photo_ is the photocurrent, I_dark_ is the dark current, and E is the incident optical power.

## 3. Results and Discussion

**Figure 2:**
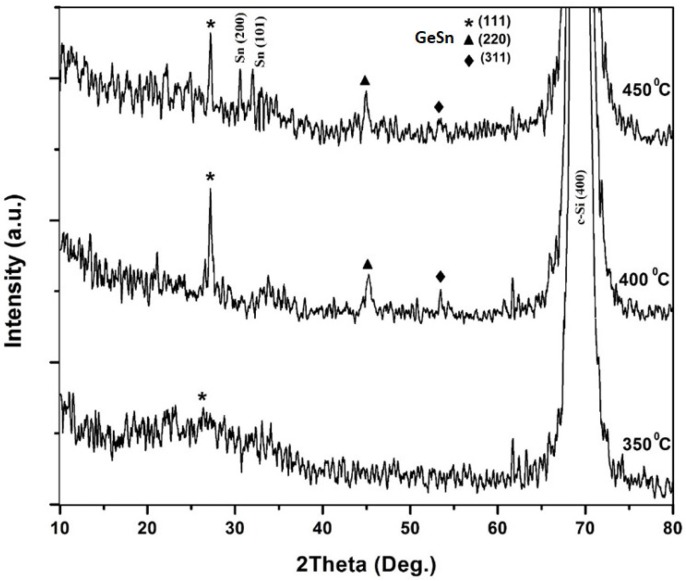
XRD pattern of the RTA samples at 350 °C, 400 °C, and 450 °C.

The HR-XRD spectra of the rapid thermal annealed multilayer of Ge/Sn/Ge, at different temperatures, are shown in Figure 2. The sharp and intense peak detected at 2θ = 69.32° is attributed to the underlying Si (400) substrate. There are no diffraction patterns for the as-grown and annealed samples, at 300 °C (not shown). The XRD results of the annealed multilayer, at various annealing temperatures, are presented in Table 1.

By raising the annealing temperatures over 400 °C, a cubic (111) diffraction pattern became pronounced, which indicated the formation of Ge_1−x_Sn_x_ alloy and an improvement in crystallinity of the annealed layers. This peak was between the 2θ angles of a-Sn (111) and c-Ge (111), 23.7°~27.3°, which is assigned to the cubic Ge_1−x_Sn_x_ (111) structure [28]. The (111) diffraction pattern of the annealed sample at 450 °C had shifted to a higher angle, compared to the heated one at 400 °C. The position of the peaks for the annealed layers, at temperatures of 400 °C and 450 °C, were detected at the diffraction angles of 27.17° and 27.22°, respectively.

Moreover, the observed peaks at 2θ = 45.20°, and 2θ = 45.25°, for the heated films at 400 °C and 450 °C, respectively, belonged to the (220) orientation between the 2θ angles of 39.2° (a-Sn) and 45.3° (c-Ge). It was detected that increasing the annealing temperature led to a small shift to a higher 2θ angle, which was a result of a reduction of the layer’s Sn content, upon heat treatment, as a result of the Sn surface segregation. Another low intensity diffraction pattern was observed at (311) orientation, between the 2θ angles of 39.2° (a-Sn (311)) and ~45.3° (c-Ge (311)), as shown in Figure 2. It was clear that the (111) diffraction pattern was stronger than the (220) and (311) peaks. The (111) planes, in a diamond cubic structure, have the closest-packed arrangement. Therefore, these planes show the lowest surface/interface free energy [29]. Consequently, the presence of a sharp (111) plane is probable, as a result of its tendency to diminish the surface/interface free energy [30]. The two other observed peaks in the annealed layer, at 450 °C, was attributed to Tin, because of surface segregation.

To estimate the average crystallite size from the full width half maximum (FWHM), Scherrer’s formula [31] was used. For the annealed layer at 450 °C, the crystallite size was higher than the one annealed at 400 °C. The sample heated at 450 °C exhibited the largest crystallite size (23 nm). The high temperature annealing leads to an increase in the mobility of the individual atoms, over the crystallite surface [32], which leads to the formation of a larger crystallite size. The reduction of the FWHM of the peaks, upon post-deposition annealing, indicated an enhancement in the average crystallite size, and consequently, an enhancement in the crystallinity of the Ge_1−x_Sn_x_ alloy films.

For estimating the Sn concentration in the annealed binary Ge_1−x_Sn_x_ films, Vegard’s law [33] was used for the 2θ angle of (111) diffraction (the shift in (220) peaks gives similar results):

(1) $$a\left(x\right)=a_{\mathrm{Ge}}\left(1-x\right)+a_{\mathrm{Sn}}x+b_{\mathrm{GeSn}}x\left(1-x\right)$$

where a is the lattice constant, x is the Sn concentration, and b_GeSn_ is the bowing parameter of GeSn (b = 0.041 Å) [34]. As given in Table 1, the estimated Sn concentration from the XRD measurements were 2.7%, 1.5% for the sample annealed at 400 °C and 450 °C, respectively. The annealed film at 450 °C showed lower Sn concentration, which suggested that a lesser amount of Sn dissolved in the Ge_1−x_Sn_x_ film. This was verified via the 2θ peak shifting to a higher angle. The synthesized Ge_1−x_Sn_x_ thin films became crystalline, at a low temperature of 400 °C, which was analogous to other research that had utilized RTA [30,35]. It was clear that the Sn incorporated in the Ge, was higher than the Sn solid solubility in Ge.

**Figure 3:**
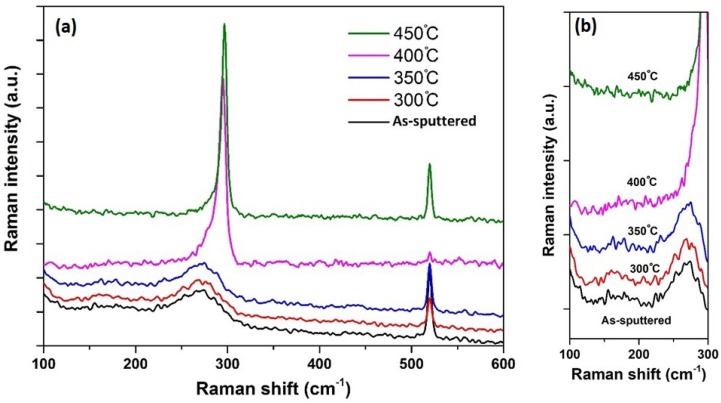
(**a**) Raman spectra of as-sputtered sample and annealed samples at 300 °C, 350 °C, 400 °C, and 450 °C. The spectra have been offset for clarity. (**b**) Sn-Sn mode of the samples.

Figure 3 displays the Raman spectra of all layers obtained at RT. Phonon peak position, intensity, FWHM, and peak shift for all layers are given in Table 2. The main sharp peak can be attributed to the Ge-Ge vibrations and the other, at 520.1 cm^−1^, belonged to the underlying Si wafer. The Ge-Sn peak was not observed. This was mainly due to the laser wavelength of 514.5 nm, used in this study, which was far from the resonance condition with the E_1_ − E_1_ + Δ_1_ optical transitions [36,37]. The Ge-Sn bond was observed when the layer was excited by higher wavelengths; like 633 nm [38,39] and 647.1 nm [37]. The Sn-Sn mode exhibited as a very low-intensity bump shape around 150 cm^−1^, as shown in Figure 3b.

Applying the heat treatment to the sputtered films made significant changes on the peak and shape of the Raman spectra. Raman results exhibited significant changes in raising the treatment temperature to 400 °C. As shown in Figure 3a, there was no significant changes in the Raman spectra of the layers annealed at 300 °C and 350 °C, compared to the as-sputtered one. However, increasing the annealing temperatures to 400 °C and 450 °C made significant changes on the Ge-Ge mode peak intensity, FWHM, and the curve shapes. This was attributed to the Ge-Sn mixing to produce the Ge_1−x_Sn_x_ alloy. Note that this crystallization temperature, at 400 °C, was lower than the one for the polycrystalline Ge [40,41] and the Si_1−x_Ge_x_ [25,26,27].

The post-deposition annealing resulted in an increase in the intensity of the Ge-Ge phonon peak, as given in Figure 3a. The layers heated at a higher annealing temperature of 400 °C and 450 °C, showed the highest phonon peak intensity, which showed intensities nearly three times higher than that of the as-deposited ones, indicating higher interactions with the incident photons, and consequently, an improvement in the crystalline structure of the Ge_1−x_Sn_x_ alloy film produced.

In addition to phonon intensity variation, the Ge-Ge peak position had shifted upon the RTA process. This peak position for the as-grown and RTA samples, at 300 °C and 350 °C, was located around 275 cm^−1^, which indicated their amorphous structure. In addition, the Ge-Ge peak in these samples belonged to the pure Ge layers, since no Ge-Sn intermixing had occurred. For the layers annealed at 400 °C and 450 °C, the Ge-Ge peak had moved to a higher frequency, which had a shift of about 18.07 cm^−1^ and 20.28 cm^−1^, respectively, compared to the as-sputtered ones. In addition, the Ge-Ge peaks of all layers were downshifted and asymmetrically broadened towards the lower frequency side, with respect to the bulk Ge peak (301.03 cm^−1^). As given in Table 2, the FWHM of the Ge-Ge peak of the resulted Ge_1−x_Sn_x_ alloy had reduced with an annealing temperature increment, demonstrating an enhancement in the crystallinity of the heated layers, at high temperatures.

Figure 3a shows the Ge-Ge peak broadening for the as-sputtered and the RTA samples, at 300 °C and 350 °C, which was due to compositional fluctuations in the alloy and the local disorder [42,43]. However, this broadening was decreased in the RTA annealed layers, at 400 °C and 500 °C, by raising the annealing temperatures, indicating the presence of a nanocrystalline-phase with good crystallinity. Note that the bulk Ge was ω_TO_ = 301.3 cm^−1^, FWHM ≈ 3.88 cm^−1^. As a result of the observed asymmetric Ge-Ge peak, the half-width at half-maximum (HWHM) were achieved for the RTA annealed samples, at 400 °C and 500 °C, as mentioned in [17,44]. The Raman spectra displayed that the left low-energy side (W_L_) was clearly larger than the right high-energy side (W_R_). Table 2 shows that the ratio of the W_L_/W_R_ was slightly decreased by increasing the heating temperature, indicating a reduction in the asymmetry and an improvement in crystallinity. As mentioned in Reference [44], the value of W_L_/W_R_ has a direct correlation with the Tin content, as was also obtained in our research. The W_L_/W_R_ ratio was higher for the annealed sample at 400 °C and lower for the annealed sample at 450 °C. It has been proposed that the detected asymmetry and the phonon peak shifting were owing to the Sn addition to the Ge matrix.

**Figure 4:**
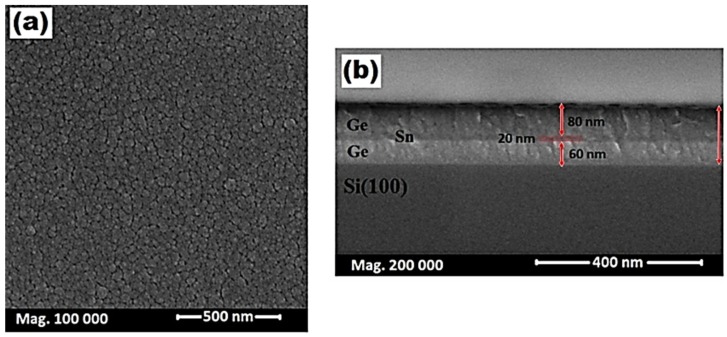
FE-SEM (**a**) planar and (**b**) cross-sectional views of the as-sputtered multilayer Ge/Sn/Ge structure.

Figure 4 shows the planar and cross-section views of the FE-SEM micrograph for the as-sputtered Ge/Sn/Ge multilayers on the Si substrate. In Figure 4a, the planar morphology of the as-grown sample displayed a densely-packed morphology. The surface contained large clusters, which resulted from the coalescence of small grains. Their range varied approximately from 15 nm to 50 nm. Figure 4b shows the cross-sectional view of the as-sputtered sample, which has clear interfaces between the Ge, Sn, and the Si. The Sn layer was embedded between the two Ge layers. The thickness of the top and the bottom layers of the Ge and Sn layers were about 80 nm, 60 nm, and 20 nm, respectively. The total thickness of the multilayer was around 160 nm from the FE-SEM figure.

**Figure 5:**
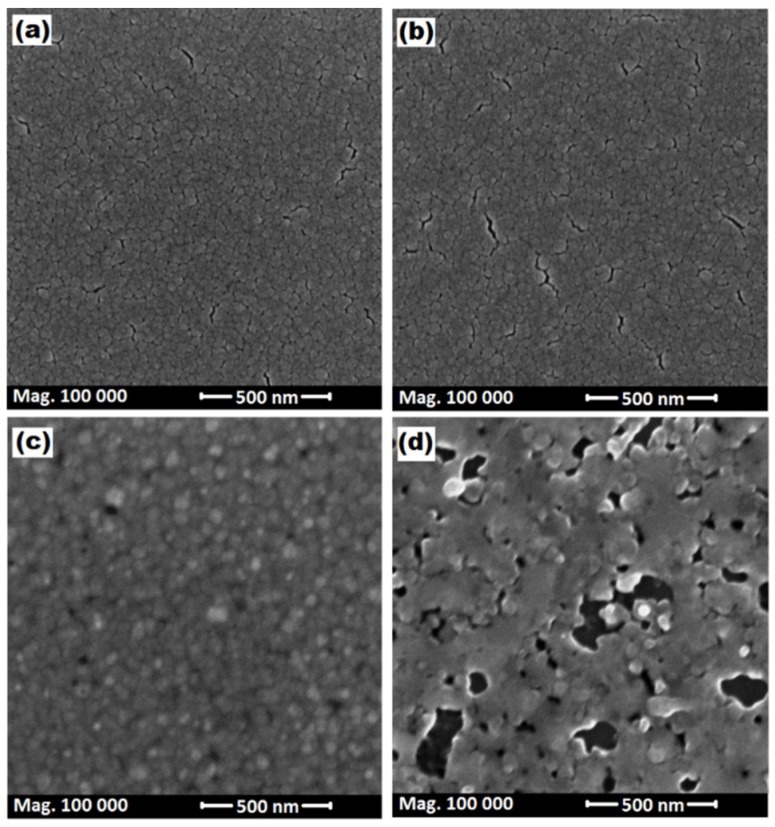
FE-SEM planar view of the RTA multilayer samples: (**a**) 300 °C; (**b**) 350 °C; (**c**) 400 °C; and (**d**) 450 °C.

Figure 5 displays the effect of the thermal annealing treatment on the multilayer Ge/Sn/Ge samples. FE-SEM images revealed quite significant changes in the surface morphology, upon RTA. However, the sample’s surface preserved the densely-packed morphology, after the annealing process. The RTA at 300 °C and 350 °C caused the appearance of fracture or nano-cracks on the film’s surface. From Figure 5c (the annealed sample at 400 °C), it is clear that the surface morphology had significantly changed, compared to the other low-temperature annealed samples. There was no sign of cracks on the surface of this sample, and the grains were slightly rounded. The film surface became more granular, with the lateral size ranging from a few tens of nanometers to about 80 nm. It was clear that that the surface aggregation was smaller at RTA of 400 °C. The reason was that the enhanced Sn atoms diffusion that was distributed on the coated flux on the layer surface, upon RTA, helped the growth of new grains and, therefore, produced a denser and finer film, which could be seen as round-shaped grains. It should be noted that the thermal expansion coefficient of Sn (23 × 10^−6^) was much higher than Ge (6.1 × 10^−6^) [45], which enhanced the solid-phase mixing. Increasing the annealing temperature, until 450 °C, led to the creation of voids on the surface of the layer, which was attributed to a greater surface segregation of Tin. These findings demonstrated the important variations in the morphology of the samples, upon RTA.

**Figure 6:**
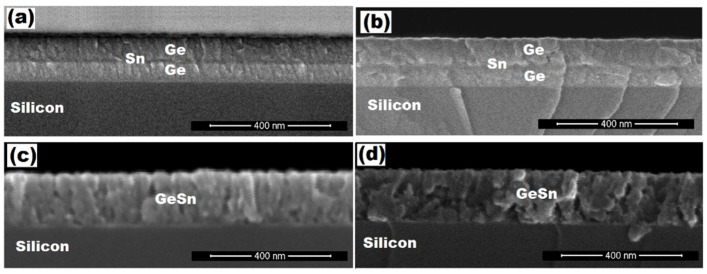
FE-SEM cross-sectional view of the rapid thermal annealed (RTA) multilayer samples: (**a**) 300 °C; (**b**) 350 °C; (**c**) 400 °C; and (**d**) 450 °C.

The cross-sectional FESEM images of multilayer structures in Figure 6 exhibited well-defined and flat heterojunction interfaces and reveals a good adhesion to the Si substrate. The cross-sectional views also reveal the Ge-Sn inter-diffusion at the annealed temperature of 400 °C and 450 °C. There is no sign of Ge and Sn layers and one single layer has been observed, which is attributed to Ge_1−x_Sn_x_ alloy film on Si substrate. While in the other samples, the Sn layer can be observed clearly. The appearance of these features indicated solid-phase mixing of Ge and Sn to form Ge_1−x_Sn_x_ alloy through inter-diffusion mechanism, which demonstrates that the incorporation of Tin into Germanium is needed to provoke the solid-phase mixing with the RTA temperature at least at 400 °C [45]. Note however, this crystallization temperature at 400 °C is much lower than the one for pure Ge, which is around 500 °C [46].

**Figure 7:**
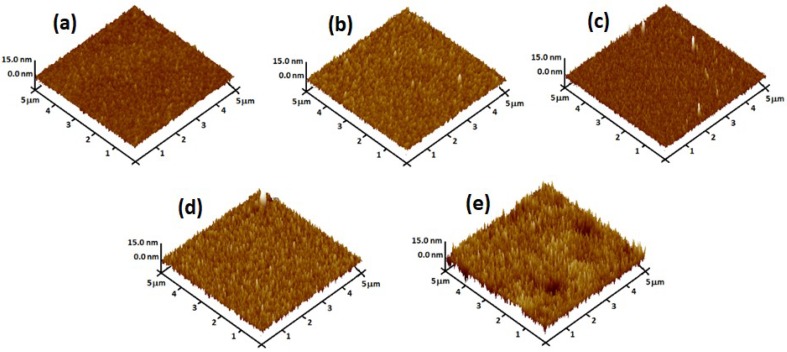
AFM images of GeSn thin films, (**a**) as-sputtered and annealed at (**b**) 300 °C; (**c**) 350 °C; (**d**) 400 °C; and (**e**) 450 °C.

The three-dimensional AFM images of the as-deposited multilayer structure and the annealed layers in a scanned area of 5 μm × 5 μm are shown in Figure 7. The root mean square (RMS) surface roughness of the films is shown in Table 3. The RMS surface of the as-sputtered multilayer sample was 1.02 nm. Significant changes were observed in the RTA annealed films at 400 °C and 450 °C, in which the RMS surface increased to 1.77 nm and 2.85 nm, respectively. The possible reason for this phenomenon was due to the solid-phase mixing (inter-diffusion) of the Ge and Sn atoms, in the multilayer structure [45], which caused the Sn surface segregation, and consequently the surface of thin films became rough, as was evident from the FE-SEM images.

**Figure 8:**
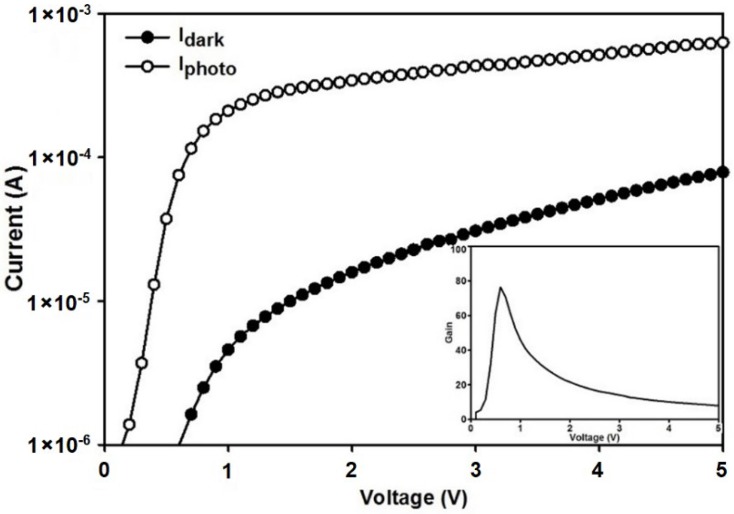
Current-voltage characteristics of the MSM PD on the RTA annealed sample, at 400 °C, measured in the dark (I_dark_), and under illumination (I_Photo_). The inset shows the current gain (I_photo_/I_dark_).

The current-voltage (I-V) characteristics of the MSM PD on the RTA annealed film, at 400 °C, is shown in Figure 8, under dark and visible light illumination. The inset shows the current gain.

**Figure 9:**
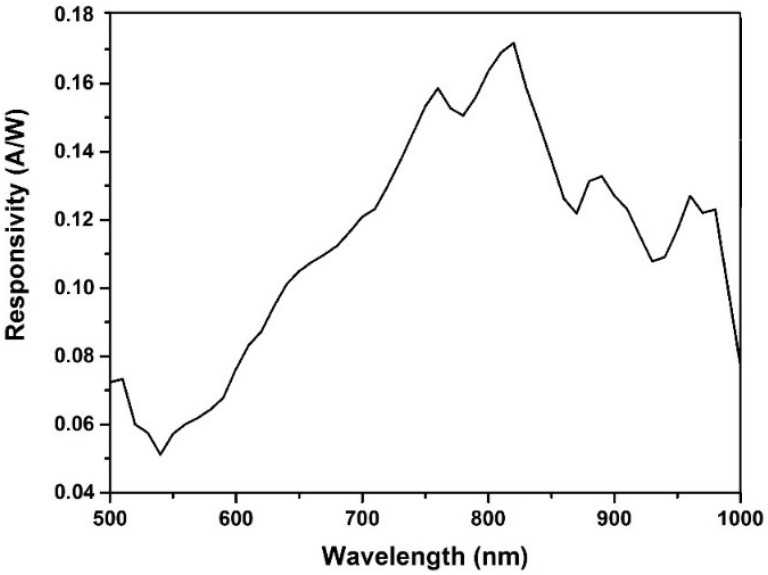
The photo-response of the Ge_0.973_Sn_0.027_ MSM PD.

The response of a Ge_1−x_Sn_x_ MSM PD increased with the bias voltage and saturated gradually at a voltage above 1.0 V, which was due to the movement of all carriers, toward the PD electrodes. It was obvious that the photocurrent produced a higher current than that of the dark, due to the photo-generation current, upon light illumination. Comparing the current at 5 V bias, the photocurrent was 5.39 × 10^−4^ A, while the dark one was 7.21 × 10^−5^ A. In addition, the current gain of this RTA sample exhibited a value of about 80, at 0.8 V bias, which indicated its higher sensitivity to the incident illumination. Meanwhile, this indicated a high photo-responsivity of the RTA Ge_1−x_Sn_x_ film. The higher current gain at this low voltage could be due to the increase of the surface resistivity. As shown in the AFM image, the RMS surface roughness of this sample (400 °C) was 1.77 nm, which was higher, compared to the as-sputtered one and moreover some small holes were observed in the FE-SEM image (Figure 5c), due to the Sn surface segregation. Therefore, this slightly high surface roughness, enhanced the resistivity of this sample and, thus, decreased the dark current value. In addition, the spectral photo-response measurement had been performed on the optimized MSM PD sample, which went through RTA at 400 °C. Figure 9 illustrates the responsivity for the Ge_0.973_Sn_0.027_ MSM PD versus the wavelength, which was measured at RT. The investigated wavelength area was from 500 nm to 1000 nm. The photocurrent value was measured at a fixed voltage of 5 V. It was clearly observed that the responsivity dropped around the 820 nm. The measured responsivity at this wavelength was ~0.17 A/W. Due to the alloy broadening, the absorption edge was not as steep as that of the pure material.

## 4. Conclusions

The nanocrystalline Ge_1−x_Sn_x_ alloy were successfully grown on the Si substrate, via Ge/Sn/Ge multilayer magnetron sputtering, after rapid thermal annealing. The Raman analysis exhibited an enhancement in the intensities of the Ge-Ge peak, upon RTA at 400 °C, and above. The XRD results demonstrated that the Ge-Sn solid-phase mixing had occurred at a low temperature of 400 °C, which led to the creation of the Ge_1−x_Sn_x_ alloy. The crystallinity of the Ge_1−x_Sn_x_ thin films improved expressively in the (111) orientation. RTA at 400 °C resulted in a uniform GeSn layer, with enhanced optical characteristics. The fabricated Ge_1−x_Sn_x_ MSM PD exhibited its photosensitivity at 820 nm. The obtained results exposed the potentiality of using the sputtering technique and the RTA to produce crystalline Ge_1−x_Sn_x_ material on the Si substrate, for photonic and light-sensing device applications.

## Figures and Tables

**Table 1:** XRD data for the annealed multilayer Ge/Sn/Ge samples at different temperatures.

Sample	Orientation	2θ (Deg.)	FWHM (Deg.)	Crystalline Size (nm)	Sn Composition (%)
400 °C	(111)	27.16	0.4208	20.30	2.7
	(220)	45.20	0.9012	9.97	
450 °C	(111)	27.22	0.3653	23.38	1.5
	(220)	45.25	0.5963	15.05	

**Table 2:** The details of the Ge-Ge mode of Raman spectra for the Ge_1−x_Sn_x_ alloy films. The frequency shift value was compared to the as-sputtered sample.

Samples	Ge-Ge (cm^−1^)	Peak Intensity (a.u.)	FWHM (cm^−1^)	Raman Shift * (cm^−1^)	Raman Shift (cm^−1^)	W_L_/W_R_
As-grown	276.32	661.00	45	24.71	-	-
300 °C	274.78	621.611	42	26.25	-	-
350 °C	274.62	659.833	35	26.41	-	-
400 °C	294.39	1773.83	12.76	6.64	18.07	3.231
450 °C	296.60	1719.00	8.29	4.43	20.28	1.274

* Raman shift compared to the bulk Ge peak (301.03 cm^−1^).

**Table 3:** Root mean square (RMS) surface roughness of the as-sputtered multilayer annealed structures and at different annealing temperatures.

Samples	As-Sputtered	300 °C	350 °C	400 °C	450 °C
Surface Roughness (nm)	1.02	1.18	1.2	1.77	2.85
